# Non-Invasive Markers for the Detection of Gastric Precancerous Conditions

**DOI:** 10.3390/cancers16122254

**Published:** 2024-06-18

**Authors:** Marcin Romańczyk, Malgorzata Osmola, Alexander Link, Amaury Druet, Caroline Hémont, Jerome Martin, Nicolas Chapelle, Tamara Matysiak-Budnik

**Affiliations:** 1 Department of Gastroenterology, Academy of Silesia, 40-555 Katowice, Poland; 2 H-T. Medical Center, 43-100 Tychy, Poland; 3 Masovian Oncological Hospital, 05-135 Warsaw, Poland; 4 Department of Gastroenterology, Hepatology and Infectious Diseases, Otto-von-Guericke University Magdeburg, Leipziger Str. 44, 39120 Magdeburg, Germany; 5 IMAD, Hepato-Gastroenterology & Digestive Oncology, University Hospital of Nantes, F-44093 Nantes, France; 6 CHU de Nantes, Laboratoire d’Immunologie, Center for ImmunoMonitoring Nantes-Atlantique (CIMNA), F-44000 Nantes, France; 7 University of Nantes, INSERM, Centre de Recherche Translationnel en Transplantation et Immunologie, UMR 1064, ITUN, F-44000 Nantes, France

**Keywords:** chronic atrophic gastritis, gastric cancer, gastric intestinal metaplasia, gastric precancerous conditions, pepsinogen

## Abstract

**Simple Summary:**

Individuals with atrophic gastritis and gastric intestinal metaplasia, considered gastric precancerous conditions (GPC), are at increased risk of developing gastric adenocarcinoma. The identification and surveillance of these patients are important for the diagnosis of early gastric neoplasia. Non-invasive markers of GPC with good diagnostic performance could allow the implementation of a stepwise screening approach and, with successful personalized endoscopic surveillance, possibly decrease gastric cancer morbidity and mortality. Pepsinogen I and II and their ratio are the most broadly investigated biomarkers with moderate diagnostic performance. Their combination with other markers like *Helicobacter pylori* antibodies and gastrin-17 (called GastroPanel^®^) allows for more precise identification of GPC but without significant improvement in overall performance. Other new serum biomarkers could possibly enhance the performance of pepsinogens. Some of them may be considered stand-alone biomarkers; however, until now, no high-quality data support the use of any of them.

**Abstract:**

Gastric cancer (GC) is still one of the most prevalent cancers worldwide, with a high mortality rate, despite improvements in diagnostic and therapeutic strategies. To diminish the GC burden, a modification of the current diagnostic paradigm, and especially endoscopic diagnosis of symptomatic individuals, is necessary. In this review article, we present a broad review and the current knowledge status on serum biomarkers, including pepsinogens, gastrin, Gastropanel^®^, autoantibodies, and novel biomarkers, allowing us to estimate the risk of gastric precancerous conditions (GPC)—atrophic gastritis and gastric intestinal metaplasia. The aim of the article is to emphasize the role of non-invasive testing in GC prevention. This comprehensive review describes the pathophysiological background of investigated biomarkers, their status and performance based on available data, as well as their clinical applicability. We point out future perspectives of non-invasive testing and possible new biomarkers opportunities.

## 1. Introduction

Gastric cancer (GC) was the leading cause of cancer death worldwide until the 1980s. Since then, GC incidence has been decreasing parallel to the decreasing prevalence of *Helicobacter pylori* (*H. pylori*) infection, the main gastric carcinogen. Despite that, currently, GC is still the fifth most frequently diagnosed cancer, responsible for almost 660,000 deaths annually, ranking as the third cause of cancer-related death in the world [1].

GC is a heterogeneous disease; different types of GC are distinguished according to their location: distal (non-cardia) GC and proximal (cardia) GC. These entities differ in terms of risk factors and epidemiological patterns. Another heterogeneity can be seen on a histopathological level. Historically, we distinguish two major subtypes according to the Laurén classification: intestinal type and diffuse type [2]. The intestinal, usually non-cardia, type is the most common (~80% of global cases), and the majority of cases are attributed to chronic *H. pylori* infection. In contrast, cardia GC has a different etiology, with only a small proportion of cases linked to *H. pylori* infection [3].

### 1.1. Gastric Precancerous Conditions 

**Figure 1:**
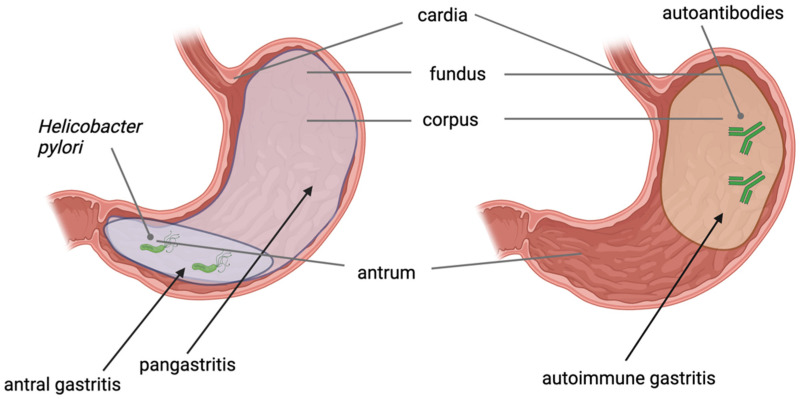
Distribution of different types of gastric precancerous conditions in the stomach. *Helicobacter pylori*-related gastritis affects the gastric antrum and eventually spreads to the corpus, causing pangastritis (on the **left**). Autoimmune gastritis affects the gastric corpus and fundus, causing mucosal atrophy that spares the antrum (on the **right**). The image was created with BioRender.com.

The development of non-cardia intestinal-type GC follows a pattern of stepwise progression beginning with gastric precancerous conditions (GPC). According to the model of gastric carcinogenesis known as “Correa’s cascade” [4], GC is preceded by a progression from a normal gastric mucosa through non-atrophic gastritis, usually induced by chronic infection with *H. pylori*, to precancerous conditions, i.e., successively, chronic atrophic gastritis (CAG), intestinal metaplasia (IM), dysplasia, and finally, adenocarcinoma [4,5,6]. Less frequently, atrophic gastritis can result from an autoimmune reaction and, in this case, is called autoimmune gastritis (AIG). In *H. pylori*-related gastritis, or non-autoimmune gastritis (NAIG), gastritis first appears in the antrum and eventually spreads to the corpus, causing pangastritis. In contrast, in AIG, atrophic gastritis is typically limited to the gastric corpus and fundus, sparing the antrum [7,8]. The distribution of gastric precancerous conditions is presented in Figure 1. 

GPC, which may be graded according to OLGA and OLGIM histological classifications, is associated with an increased risk of GC [9]. The annual incidence of GC in patients with GPC, according to a PALGA study conducted on the Dutch population, was 0.1% for CAG, 0.25% for IM, 0.6% for mild-to-moderate dysplasia, and 6% for severe dysplasia (for the latter, HR 40.14, 95% CI; 32.2–50.1) [10]. Studies have demonstrated that the most common location of gastric atrophy is the antrum, but patients with pangastritis have a major risk of progression to GC [11]. To sum up, patients with CAG have an increased risk of GC; therefore, they should benefit from close surveillance [12].

### 1.2. Screening Programs and Preventive Measures for Gastric Cancer

Up to now, GC screening programs have been implemented in countries with a high incidence of GC (e.g., Japan, South Korea, and China) to enable the diagnosis at the earlier stage and improve survival. So far, there are no established screening programs for GC in Europe. However, there are currently ongoing European projects (GISTAR: Multicenter randomized study of *Helicobacter pylori* eradication and pepsinogen testing for prevention of gastric cancer mortality; EUROHELICAN: Accelerating gastric cancer reduction in Europe through *Helicobacter pylori* eradication, TOGAS: TOwards GAstric cancer Screening Implementation in the European Union) aiming at the evaluation of feasibility and the most appropriate modalities of screening programs in different European countries. In these projects, different potential approaches are being compared, including systematic screening for *H. pylori* infection in the young population, pepsinogen serology followed by upper endoscopy in case of pathological result, or the upper endoscopy systematically added to the screening colonoscopy [13]. In another ongoing European project, AIDA (Artificially Intelligent Diagnostic Assistant for gastric cancer inflammation), even the possibility of creating an artificial intelligence tool to better evaluate the risk of the evolution of GPC towards gastric cancer will be evaluated. Since most GC cases progress from GPC, several actions have been proposed to reinforce the surveillance of patients with GPC. One of them consists of open-access endoscopy services in patients with high-risk GPC [10,14]. A combined screening colonoscopy and esophagogastroduodenoscopy has also been proposed as combined colon and gastric cancer screening [15]. However, the endoscopic diagnostic performance of gastric premalignant conditions—CAG and IM—is questionable. The real-world data show that the sensitivity of the detection of CAG does not exceed 70% and the detection of IM 20% [16,17]. The diagnostic performance depends on the operator’s expertise and may vary significantly between centers [17,18]. Because of low detection by optical judgment, the diagnosis of CAG and IM still relies on “mapping” biopsies [12]. On the other hand, endoscopically diagnosed atrophy is a well-established risk factor for GC [19]. Risk estimation based on endoscopy may be based on the solitary endoscopic diagnosis of severe atrophy or confirmation of IM at biopsies [20]. The endoscopic grading of the extent of mucosal atrophy, called the Kimura–Takemoto classification, was introduced over 50 years ago [21]. Recently, important progress has been made in the endoscopic diagnosis of gastric atrophy and intestinal metaplasia thanks to the introduction of high-performance endoscopy techniques, including high-resolution, magnifying endoscopy, and virtual chromoendoscopy [22]. The endoscopic grading of gastric intestinal metaplasia (EGGIM) is a precise parameter of gastric IM estimation [23,24]. It is also worth mentioning that the endoscopic evaluation of IM shows promising results in the identification of an incomplete type of IM known for a greater risk of GC [25]. Nevertheless, the endoscopic evaluation of pre-malignant conditions is imperfect as a screening measure. Despite the low rate of adverse events, esophagogastroduodenoscopy (EGD) is an invasive and costly diagnostic procedure with reported complications [26]. The estimated number of procedures for one cancer avoided by the detection of a premalignant condition exceeds 230 in countries with low to intermediate prevalence of GC [27]. Also, non-invasive screening might potentially be a complementary alternative to screening EGD, as not all of the population would be willing to undergo this procedure [28]. Therefore, the development of non-invasive markers is required to “support” or replace endoscopy in searching for pre-malignant conditions. It would be applicable, especially in countries with low to moderate GC incidence, where nationwide screening programs regarding cost-effectiveness and burden of patients seem not appropriate. 

### 1.3. Blood Biomarkers

#### 1.3.1. Validated and Commercially Available Biomarkers of Gastric Atrophy

##### Pepsinogens—PG I and PG II Blood Concentration

Serum pepsinogens (PGs), the precursors of pepsin, are the most studied biomarkers of gastric atrophy. PGs include PGI and PG II, secreted in the stomach lumen and circulation. PGI is secreted by the chief cells present only in the gastric corpus, while PGII is secreted throughout the stomach and proximal duodenum. Therefore, in the case of atrophic gastritis affecting the corpus, the level of PGI drops significantly. In contrast, the level of PGII remains unchanged, hence allowing the use of the decreased levels of PGI and PGI/PGII ratio as potential biomarkers of corpus atrophy. One of the weaknesses of the non-invasive diagnosis of CAG using PG testing is its poor performance in the detection of antrum atrophy. However, it appears as a good marker of corpus and especially marked atrophy [29]. Moreover, some recent data show that PGI testing may be useful in distinguishing the cause of CAG, its levels being significantly lower in autoimmune gastritis, than in *Helicobacter pylori*-induced CAG [30].

The diagnostic value of PG testing has been assessed in several studies using different methods (enzyme-linked immunosorbent assay, ELISA; Chemiluminescent Immunoassay, CLEIA), with a different cut-off value for each method and adjusted for different population/ethnicity [30,31,32,33,34,35,36,37,38] (Table 1). 

The role of PG serology in atrophic gastritis has been underlined in several international recommendations: MAPS I and II consensus stated that serum pepsinogen levels can predict extensive CAG. Also, low PGI serum levels or/and a low PGI/II ratio identify patients with advanced stages of atrophic gastritis, and endoscopy is recommended for these patients, particularly if *H. pylori* serology is negative (high-quality evidence, strong recommendation [12,50]). In the Maastricht VI/Florence Consensus, the role of PG testing has been discussed. It has been emphasized that PG testing is one of the screening modalities in countries with intermediate risk of GC. The PG levels help to estimate the risk of atrophy and differentiate the etiology of atrophic gastritis [30,51]. The Kyoto consensus confirmed that serological tests (PGI, PGII, and *H. pylori* antibody) are useful for identifying individuals at increased risk for GC (grade of recommendation strong; high level of evidence [52]). It is worth mentioning that according to MAPS II guidelines, only individuals with stages III and IV of OLGA and OLGIM should be assigned for surveillance [12]. It was shown that the performance of PGI and PGI/II testing is rising with the severity of CAG [31,34,43]. Presented outcomes reflect a diagnosis of CAG based on histopathological examination. Still, PGI and PGI/II also showed similar outcomes in the endoscopic diagnosis of atrophy using the Kimura–Takemoto classification [32,37,39]. Also, PG testing is effective in the prediction of atrophy based on the origin of gastritis. Along with the mentioned data regarding PGI concentration, the PGI/II ratio was also lower in autoimmune CAG, with better testing performance [30,33]. It is an essential issue as patients with autoimmune CAG are believed to have a greater progression risk to cancer [53,54].

Despite the mentioned recommendations, the performance of PG testing varies significantly among presented studies (Table 1). Different outcomes of study results might be related to the differences in studied populations. Indeed, it has been shown that the serum concentration of the pepsinogens and PGI/II ratio may vary between countries, even among one geographical region [45]. Furthermore, the selection of patients for the studies, bearing different types (in terms of their origin and localization) of CAG, as well as different levels of severity, may be responsible for the discrepancies in the results obtained. Therefore, studies on well-characterized groups of patients are important to help better define the diagnostic value of PG testing [30].

To achieve better testing performance, in recent studies, PGI and PGI/II ratios have been combined with various other markers, such as human epididymal protein 4 (HE4), interleukin-5, or miR-101-3p, to achieve better diagnostic performance [31,35,44]. The highest reported area under the curve (AUC) was 0.917, with 95.1% sensitivity and 80% specificity in the diagnosis of CAG compared to controls for a combination of PGI and PGI/II ratio with miR-101-3p [35].

Altogether, PG testing appears as a sensitive method to detect corpus atrophy, especially in the context of AIG, but is a poor marker for the detection of antrum atrophy.

##### Gastrin

Gastrin is produced by gastric G cells located in the gastric antrum. Gastrin stimulates the release of gastric acid in the stomach after food intake. Its secretion is regulated by a feedback system involving (i) the presence of peptides in the stomach, (ii) high pH in the stomach, and (iii) positive regulation by gastrin-releasing peptide, acting together with a negative regulation by somatostatin [55]. Gastrin is translated as a 101-amino-acid precursor which undergoes processing to the products gastrin 17 and gastrin 34 [55]. However, only the gastrin 17 (G17) measurement is used in clinical practice [56]. Gastrin production increases after food intake; therefore, evaluating G17 following a protein-rich meal is more accurate than fasting gastrin [57].

In AIG, reduction in gastric acid secretion triggers a compensatory response, resulting in an increase in gastrin levels that stimulates the release of gastric acid from parietal cells. Therefore, increased G17 is a good serological marker of AIG [58]. Some studies reported that gastrin levels are higher (~1.5-fold) in patients with *H. pylori* infection than in uninfected patients and long-term proton pump inhibitor users [59]. In the CAG of the antrum, the loss of antral glands may result in a decreased number of G cells, leading to a low output of G17. Therefore, a low G17 level could be a marker of gastric antral atrophy. Some previous studies evaluated the diagnostic value of gastrin in this indication; the test’s sensitivity was 36.8%, specificity 86.5%, and the overall accuracy 82.6% after protein-meal stimulation [57].

In the guidelines, G17 is currently recommended by Maastricht VI/ Florence guidelines as a part of the assessment of gastric functional serology, together with PGs and anti-*H. pylori* antibodies, anti-intrinsic factor, and anti-parietal cell autoantibodies. G17 provides clinically valuable information on the likelihood of gastric mucosal atrophy, including its etiology (grade A1 recommendation) [51].

Overall, the low sensitivity of the G-17 test made it less useful for diagnosing antral atrophy in clinical practice as the sole marker. Nevertheless, there is an additional gain of adding G17 to the PG as a biomarker, notably as a part of Gastropanel^®^ [43].

##### Gastropanel^®^

GastroPanel^®^ is a combination of serological assays, including serum PGs (PGI and PGII), G-17, and anti-*H. pylori* antibodies (HpAb), which has been proposed as a ‘serological biopsy’ for the diagnosis of CAG [60,61]. The interplay of interdependent biomarkers measured in serum samples can help to assess the presence of CAG and the activity of gastric inflammation in the antrum and corpus separately. Serum PGI levels and/or the PGI/PGII ratio are low in patients with corpus and fundus CAG. The G17 level is high in CAG limited to the corpus and fundus but low or non-elevated if the CAG occurs in both the antrum and corpus [62]. Therefore, a low G17 serum level in combination with positive HpAb would indicate the presence of antrum atrophic gastritis related to *H. pylori* infection. Thus, combining the results of the HpAb, PGI, or PGI/PGII ratios and G17 tests would allow for detecting the presence and site of inflammation [63].

A meta-analysis performed by Zagari et al. included 20 studies assessing the accuracy of a combination of serological assays (PGI, PGI/PGII ratio, G17, *H. pylori* serology) for the diagnosis of CAG, compared to histology. Pooling data from these studies yielded a summary sensitivity of 74.7% (95% CI; 62–84.3) and a specificity of 95.6% (95% CI; 92.6–97.4). Based on the median prevalence of CAG across the studies of 27%, the negative predictive value of the panel test was 91%, and the positive predictive value was 86% [60]. Although the demonstrated sensitivity for GastroPanel^®^ is very high, we need to take into consideration that there is a wide heterogeneity of the results obtained in the studies included in this meta-analysis, with the sensitivity ranging between 32 and 98% [60]. These data seem to be supported by another recent meta-analysis where diagnostic performances were good for the estimation of corpus CAG [64]. In a recent study based on a population screening in Taiwan, GastroPanel^®^ showed a high sensitivity exceeding 80% but with a low specificity of 48.8% (95% CI 42.5–55.0%) [65]. It is also worth mentioning that the performance of the Gastropanel^®^ test based on ELISA analysis is comparable with LZ-Test^®^, where the same components have been analyzed with a latex-enhanced turbidimetric immunoassay (L-TIA) [66]. In the study performed by Chapelle et al., sensitivity, specificity, positive, and negative predictive values for the detection of CAG by GastroPanel^®^ in a European population were 39.9% (95% CI 31.9–48.2), 93.4% (88.9–96.4), 81.9% (71.1–90), and 67.3% (61.4–72.8), respectively. The sensitivity was significantly higher for the detection of severe CAG [60.8% (95% CI 46.1–74.6) *p* = 0.015] and corpus CAG [61% (49.2–72), *p* = 0.004] [43]. The better performance of GastroPanel^®^ in the prediction of corpus CAG has been underlined by another meta-analysis where pooled sensitivity and specificity for corpus and antrum atrophy were 70.2% and 51.6% for sensitivity and 93.9% and 84.1% for specificity of corpus and antrum, respectively [67]—see Table 2. However, studies by Chapelle and McNicholl showed that diagnostic performances of GastroPanel^®^ were not significantly better than PGI alone [43,68]. Also, the recent data showed the limited performance of AUC: 0.62 in the detection of CAG and IM in an American population [41]. It should be mentioned that, in a study with a limited number of patients, GastroPanel^®^ was not shown to be valuable for AG diagnosis [69]. On the other hand, data on a larger number of patients (n = 512) gave an overall agreement of GastroPanel^®^ and updated Sydney system exceeding 90% [29].

Recently, another important issue has been raised. GastroPanel^®^ testing could be not only a mass screening tool for GPC but also an effective triage tool in the referral for gastroscopy decision-making [70]. Also, combined PG and gastrin levels as part of the prediction model could be helpful in the identification of IM or dysplasia among individuals with CAG [71]. It can also be helpful in the identification of autoimmune gastritis among patients with CAG with or without IM [72].

**Table 2:** Studies describing the accuracy of GastroPanel^®^ in the diagnosis of atrophic gastritis.

Study (Year)	Study Type, Country	Targeted Condition	Cut-Off Value	Test Method	No. of Patients Included	Age, Mean ± SD (Range)	Sensitivity % (95% CI)	Specificity % (95% CI)
Dondov [73] (2022)	Single center, Mongolia	AG, GC	PG I≤ 75.07 ng/mL, PGI/II ratio ≤ 6.25, G-17 ≤ 23.42 pmol/L	ELISA	114 (AG: 40, GC: 36)	59.98 ± 10.88	80.0	60.5
Syrjänen [64] 2022	Meta-analysis, 49 studies	corpus AG	PG I lower than threshold, PG I/II lower than threshold, G-17 higher than threshold	different	22 597	n/a	70 (64–76)	93 (90–95)
Chiang [65] (2020)	Multicenter (population-based screening), Taiwan	AG	PG I < 30 μg/L; PGI/PGII ratio < 3 G17b < 1; HpAb < 30 *	ELISA	465	n/a	80.6 (70.0–88.3)	48.8 (42.5–55.0)
Chapelle [43] (2020)	Multicenter, France	AG	PG I < 30 μg/L; PGI/PGII ratio < 3 G17b < 1; HpAb < 30	ELISA	344 (AG: 148)	58.8 ± 14.2	39.9 (31.9–48.2)	93.4 (88.9–96.4)
Mattar [46] (2020)	Single center, Brasil	AG		n/a	308 (AG: 135)	64.6 ± 10.3		
Zagari [60] (2017) **	Meta-analysis of 20 studies	AG	PGI;PGI/PGI/II ratio; G17b; HpAb; different cut-offs	ELISA	4241	n/a	74.7 (62.0–84.3)	95.6 (92.6–97.4)
Syrjänen [67] (2016)	Meta-analysis of 27 studies	corpus AG	PGI;PGI/PGI/II ratio; G17b; HpAb; cut-offs n/a	n/a	8654	n/a	70.2 (64.3–77.5)	93.9 (91.0–96.0)
		antrum AG					53.8 (38.3–68.7)	84.1 (71.3–91.9)
McNicholl [68] (2014)	Multicenter, Spain	AG	PGI < 25 μg/L G-17b < 0.1 HpAb < 30	n/a	85	44	50 (39–61)	80 (71–88)

CI, confidence interval; ELISA, enzyme-linked immunosorbent assay; AG, atrophic gastritis; HpAb, antibodies to *H. pylori* [EIU]; EIU, enzyme immune units; PGI, pepsinogen I; PGII, pepsinogen II; G-17b, basal; Gastrin-17 [pmol/L]; * cut-off values not clearly defined, results based on the GastroSoft^®^ ** in the study PGI, Gastrin 17, *Helicobacter pylori* with or without PGI/PGI ratio were analyzed.

#### 1.3.2. Other Potential Biomarkers

##### MicroRNAs and Long Non-Coding RNA Polymorphism

Non-coding RNA (ncRNA) are among the most novel molecules in cellular physiology, and despite the enormous scientific interest, their translational impact on clinical practice remains to be determined. There are different subtypes of ncRNA that may play a substantial role in pathophysiology or have a potential diagnostic role in the identification and management of preneoplastic conditions and include microRNAs (miRNAs) [74], long non-coding RNA (lncRNA) [75], xeno-miRNAs (exogenous miRNAs) [76], or circular RNA (circRNA) [77]. However, despite the heterogeneity of their forms, they have several things in common, including interference with gene expression and, therefore, substantial involvement in cellular processes such as cell proliferation and apoptosis, as well as in carcinogenesis [78,79,80]. The deregulation of miRNA has been repeatedly demonstrated in GC, and alterations of miRNA have also been reported in preneoplastic conditions [81,82]. A study by Marquez et al. showed that the expression levels of inflammatory miRNAs—miRNA-146a and miRNA-155—increased by an average of 21- and 62-fold, respectively, in adult patients with gastritis compared to the controls [82]. In one of the first studies where miRNA expression has been systematically evaluated, the differential expression of miR-21, miR-155, and miR-223 was reported in patients with preneoplastic conditions and strongly linked to *H. pylori* infections but not to PPI or aspirin use [81,83]. Additionally, the expression levels of miRNA may be *H. pylori* strain-specific, with a link to virulent *H. pylori* strains, indicating its potential role in the pathogenesis of *H. pylori*-associated gastritis and likely initiation of the early neoplastic cascade [84]. However, not only miRNA expression but also the CpG Island methylation of miRNA genes or single-nucleotide polymorphisms (SNPs) may be relevant as potential biomarkers [85]. In a study performed by Okubo, patients with certain SNPs in miRNAs had an increased susceptibility to GC [86]. The role of Epstein–Barr Virus-encoded miRNA has also been linked to gastric carcinogenesis and its associated molecular alterations [87,88,89]. Similarly, alterations in lncRNA HOX transcript antisense RNA (HOTAIR) have been reported not only in GC but, most interestingly, in GPC, specifically intestinal metaplasia, suggesting its role in early carcinogenesis [90].

To summarize, there is a great hope that miRNA, as a unique biomarker with extraordinary stability against degradation, may facilitate the early identification of patients at risk for GC development, but the use of tissues may only extend the current invasive practice, including upper GI endoscopy or histological assessment. Therefore, there is a great need for non-invasive biomarkers. A comprehensive systematic review of miRNA as non-invasive biomarkers for gastric cancer found that although miRNA-based tools may be a promising tool for non-invasive diagnosis of GC, there are still significant concerns that need to be addressed in future studies [91]. In particular, the quality of the reports is quite heterogeneous, and many studies are still underpowered due to a lack of independent validation [92]. Furthermore, technical comparisons are currently difficult, and the use of non-microRNA-based techniques for normalization must be interpreted with caution. Most importantly, there is still limited data on miRNA as non-invasive biomarkers, which needs to be addressed in the future.

## 2. Hormones

### 2.1. Leptin

Leptin is a peptide hormone that plays a key role in appetite and metabolism regulation. It is primarily produced by adipocytes but also in the gastrointestinal tract by gastric chief cells, along with PG I. Leptin receptors (OB-R) are present in the stomach and intestines. Thus, the stomach is a unique organ which expresses both leptin and its receptors and transduces autocrine leptin signaling [93,94]. Increased leptin levels act as a pro-inflammatory stimulus for various organs [95]. In murine models, increased gastric leptin levels with microbial dysbiosis lead to the development of IM in the stomach [94,96].

Leptin serum levels are increased in the presence of GPC [97,98,99]. In a study performed by Capelle et al., serum leptin was higher in patients with IM or dysplasia than in healthy controls [98]. The immunohistochemical examination of the stomach tissue showed that leptin and OB-R expression were higher in the presence of GPC [100]. The expression of leptin in the GC tissue was significantly associated with advanced stage, poor tumor differentiation, and risk of metastasis [101,102].

Although serum leptin levels are of significant additional value in predicting gastric IM, measuring leptin levels does not seem very useful in clinical practice for screening purposes in patients at risk for GC due to the high variability of leptin and a variety of factors influencing its levels such as age, body mass index (BMI), sex (female > male), smoking, and the distribution of adipocytes [98,99].

### 2.2. Adiponectin

Adiponectin (APN) is produced by adipocytes and plays an important role in energy metabolism and insulin sensitivity. APN is inversely related to BMI and may modulate obesity-related malignancies [103]. In precancerous conditions such as CAG as well as in *H. pylori* infection, APN can be used to identify patients at risk of developing metabolic syndrome [104,105]. Adiponectin may enhance carcinogenesis through its well-recognized effects on insulin resistance as well as its direct effect on tumor cells [106]. A study by Ishikawa et al. suggested that serum APN is lower in patients with GC as compared to healthy controls [107], but in a study performed by Seker et al., there was no statistical significance between the groups [108]. Additionally, as in the case of leptin, serum APN levels may vary due to multiple factors (sex, body fat distribution, renal and cardiac function, smoking, dietary factors, and physical exercise [106]), making it difficult to implement in clinical practice to diagnose GPC.

### 2.3. Insulin-like Growth Factor

The insulin-like growth factor (IGF-)1 is a peptide hormone secreted primarily by the liver. The IGF-1 signaling system plays a central role in cellular growth, differentiation, and proliferation and may act as an oncogene [109,110,111].

Patients with GC have increased levels of growth factors, including IGF, as compared to healthy individuals [112]. The study performed by Ennishi et al. shows that certain IGF1 genetic variations are significantly associated with GC risk in the Japanese population [113]. Another study found an increased expression of IGF 1 and 2 receptor genes (at the mRNA and protein level) in GC when compared with non-tumor tissue. These findings suggest that IGF is involved in the pathogenesis of GC, probably by autocrine/paracrine stimulation of cell growth. [114]. The eradication of *H. pylori* leads to a mild but statistically significant decrease in serum IGF-1 levels, which may be due to a decrease in antral inflammation and the inhibitory effects of eradication regimes on a synthesis of IGF-1 by the liver [115]. Despite IGF’s important role in GC carcinogenesis, the role of IGF in the development of GPC is still unknown. Also, IGF is primarily produced by the liver, not by gastric tissue, which makes it difficult to make the assessment relatable to the gastric mucosa. Therefore, the role of IGF as a marker of GPC is limited.

## 3. Autoantibodies

Recent epidemiological studies on GC have shown a rising incidence in young, especially female patients [116,117]. The causal mechanism for this “new” type of GC has not been identified. However, a role for autoimmunity or changes in the microbiota has been proposed [117,118,119]. This is supported by studies suggesting an association between autoimmune conditions, such as dermatomyositis, pernicious anemia, Addison disease, and herpetiform dermatitis, and an increased risk of GC [120,121,122]. In the recent meta-analysis by Song et al., an autoimmune condition was associated with a GC pooled relative risk (RR) of 1.37 (95% CI, 1.24 to 1.52). Among the 24 autoimmune conditions, two autoimmune diseases were mainly associated with increased risk of GC: dermatomyositis (RR, 3.69; 95% CI, 1.74 to 7.79) and pernicious anemia (RR, 2.84; 95% CI, 2.30 to 3.50) [120]. If autoimmunity is associated with the development of GC, one would expect the presence of a biological stigma of autoimmunity in patients with GPC, which precedes the appearance of cancer.

In the study by Osmola et al. that evaluated the frequency of autoantibodies (anti-nuclear antibodies (ANA), anti-parietal cell antibodies (APCA), anti-intrinsic factor antibodies (AIFA), and 16 myositis-associated antibodies) as potential markers of GPC, showed that ANA positivity was significantly higher in AIG than in *H. pylori*-related gastritis or in control patients (46.7%, 29%, and 27%, respectively), but the clinical significance of this finding remains to be established [123]. Myositis-associated antibodies were not higher in patients with GPC compared to the control group. Higher APCA and AIFA positivity was confirmed in AIG, whereas *H. pylori* infection does not affect autoantibody seropositivity (ANA, APCA, AIFA) [123]. In contrast, other studies suggest a link between higher APCA and AIFA autoantibodies and *H. pylori* infection [124,125]. Overall, the autoantibodies alone do not appear as good markers of GPC [123].

## 4. Other Potential Biomarkers

Due to the high frequency of GC, the search for new biomarkers of GPC is under investigation to improve the diagnostic performance of PG.

### 4.1. Human Epididymal Protein 4

Increased serum level of human epididymal protein 4 (HE-4) is an ovarian cancer biomarker established in the clinical guidelines. HE-4 is upregulated in GPC in the metaplastic transition following acute parietal cell loss in mice and humans and has been suggested as a surrogate marker of preneoplastic conditions in the stomach [126]. HE-4 can also be expressed by GC—the expression in immunohistochemical examination was present in 25% of intestinal type and around 60% of diffuse type GC of stages I and II; its expression correlated with tumor size, stage, and survival [127,128]. HE-4 expression was also present in other gastrointestinal cancers, like pancreatic and esophageal cancer [127]. In a study performed by Chapelle et al., combining the PGI/II ratio with serum HE-4 concentration showed an increased sensitivity of up to 85.2% for detecting moderate to severe atrophic gastritis at any location, whereas the PGI/II ratio alone demonstrated 75% sensitivity and 92.6% specificity for the detection of moderate to severe corpus CAG [31]. Therefore, HE-4 can be a good additional marker for the diagnosis of GPC.

### 4.2. Interleukin-6

Interleukin-6 (IL-6) is a pleiotropic cytokine that plays a role in inflammation and tumor progression. Recent studies have shown that *H. pylori* induces signal transduction and activation of transcription 3 (STAT3), which plays a vital role in gastric carcinogenesis. STAT3 activation is mediated through reactive oxygen species (ROS)-induced upregulation in IL-6 expression in human GC cells [129]. These findings provide a novel molecular mechanism responsible for *H. pylori*-induced gastritis and gastric carcinogenesis and a possibility to use serum IL-6 as a GPC biomarker. Additionally, higher IL-6 serum levels were detected in *H. pylori*-infected individuals [130]. Increased levels of IL-6 and other chemokines have been associated with GC growth, and IL-6 serum levels increase during tumor progression and correlate with patient survival. Several studies have investigated the IL-6 value as a diagnostic marker of GC, with a range of sensitivity and specificity of 0.39–0.85 and 0.50–0.97 [131,132,133]. Of note, IL-6 values may be influenced by other factors, including autoimmune diseases, inflammation, and physical exercise, and thus, this parameter is susceptible to giving false-positive results. In a study performed by Chapelle et al., IL-6 showed a promising sensitivity of 72.2% for the detection of antral CAG, which makes it an interesting marker for detecting atrophy in this location [31].

## 5. Conclusions and Future Directions

The approaches described in this review are supposed to offer a new diagnostic possibility for GPC and, as an effect, help decrease GC mortality in the near future. We should keep in mind that according to the WHO, markers, to become screening tests, must fulfill certain criteria [134]: (i) The diagnosed condition should be an important health problem. As GPCs are formed ahead of GC, their diagnosis and surveillance are important to closely monitor people who are at risk of developing GC and eventually treat the disease at the early stage. (ii) The test must be safe to administer and acceptable to the population. While endoscopy is an invasive test and not well accepted by patients, we should consider embracing the development of noninvasive markers to improve the adherence of patients. (iii) The test should be reasonable in terms of cost. Tests should be affordable to be accepted by health systems, especially in low- and middle-income countries, where most of the GC cases occur. (iv) Tests should lead to the improvement of health outcomes. By implementing screening tests for GPC, we can, in consequence, decrease the mortality of GC. (v) Test should be sensitive. High sensitivity with high specificity is the main goal of screening tests. To date, different molecular targets have been analyzed as potential biomarkers in patients at risk of GC. However, the single biomarker with sufficiently high sensitivity and specificity to be used in broad routine practice has yet to be identified or validated. Pepsinogens are the most studied biomarkers, and potentially combining pepsinogens with one or more of the emerging tests could potentially improve the performance of non-invasive testing for GPC. Despite all the progress made in the field of non-invasive markers of GPC, it is important to stress that, for now, careful endoscopic examination with gastric biopsies for histological assessment remains the most reliable method for GPC detection and grading.

## Figures and Tables

**Table 1:** Studies describing the accuracy of pepsinogen levels in the diagnosis of atrophic gastritis.

Study (Year)	Study Type, Country	Targeted Condition	Cut-Off Values	Test Method	No. of Patients Included	Age, Mean ± SD (Range) Years	Sensitivity % (95% CI)	Specificity % (95% CI)	AUC ROC (95% CI)
Lin [39] (2023)	Single center, China	AG	PG I ≤ 70 ng/mL, PGI/PGII ratio ≤ 3	n/a	965 (AG: 275)	n/a	8.7	94.5	n/a
			PG II > 11.05 ng/mL, PGI/PGII ratio < 3.75				21.8	86.1	n/a
Sivandzadeh [40] (2023)	Single center, Iran	AG	PG II > 30.28 μg/L for corpus atrophy	ELISA	153	63.7 female; 64.9 male	28.6 (8.6–58.1)	93.5 (88.1–97.0)	n/a
			PG I for corpus atrophy				n/a	n/a	0.551
			PGI/II ratio for corpus atrophy				n/a	n/a	0.544
Chapelle [31] (2022)	Multicenter, France	AG	PGI ≤ 21.1 ng/mL	CLEIA	356 (AG: 152)	58.6 ± 14.2	40.8 (32.9–49.0)	94.6 (90.6–97.3)	0.642
			PGI/PGII ratio ≤ 3.03				46.7 (38.6–55.0)	92.6 (88.2–95.8)	0.685
Huang [41] 2022	Single center, United States of America	AG, IM	PGI < 67 μg/L	ELISA	135 (AG or IM: 59)	n/a	41 (29–54)	78 (68–87)	0.567
			PGI/PGII ratio < 8.2				43 (30–56)	45 (34–56)	0.503
Nguyen [32] (2022)	Single center, Vietnam	AG moderate to severe	PGI ≤ 63.5 ng/mL	CMIA	273 (moderate to severe AG: 77)	56.3 ± 9.7	79.2	41.3	0.612
			PGI/PGII ratio ≤ 5.2				61	68.9	0.689
			PGI ≤ 63.5 ng/mL, PGI/PGII ratio ≤5.2				49.4	82.1	n/a
			PGI ≤ 63.5 ng/mL, PGI/PGII ratio ≤ 5.2				90.9	28.1	n/a
Miftahussurur [42] (2022)	Multicenter, Indonesia	AG, GC, gastroesophageal reflux	PG I ≤ 70 ng/mL, PGI/PGII ratio ≤ 3	ELISA	646 (AG: 171)	44.93 ± 12.98	7.6 (4.5–9.2)	99.2 (98.2–99.8)	n/a
			PGII ≥ 12.45 ng/mL		646 (AG: 27)		59.3 (38.8–77.6)	77.1 (73.0–80.8)	0.755 (0.702–0.811)
			PGI/II ratio ≤ 4.75				81.5 (61.9–93.7)	78.7 (74.3–82.3)	0.821 (0.763–0.855)
Koc [33] (2022)	Single center, Turkey	AG, autoimmune AG	PGI/II ratio ≤ 11.9 for AG and autoimmune AG	ELISA	147 (AG:79, autoimmune AG: 16)	57.7 ± 12	45.6	84.4	0.644
			PGI/II ratio ≤ 9.2 for AG				47.5	90.6	0.711
			PGI/II ratio ≤ 1.9 for autoimmune AG				100	100	1
			PGI ≤ 13.5 ng/mL for autoimmune AG				100	100	1
Cai [34] (2021)	Multicenter, China	AG	PGI ≤ 73.14 ng/mL OLGA 0 versus I/II	CLIA	1922 (OLGA 0: 1590, Olga I/II: 273, OLGA III/IV: 49)	52.3 ± 9.8	62.1	53.8	0.585
			PGI/PGII ratio ≤ 11.54 ng/mL OLGA 0 versus I/II				43.2	77.7	0.611
			PGI ≤ 64.0 ng/mL OLGA 0/I/II versus III/IV				67.2	61.2	0.631
			PGI/PGII ratio ≤ 9.11 ng/mL OLGA 0/I/II versus III/IV				53.0	91.8	0.740
Chapelle [43] (2020)	Multicenter, France	AG	PG I < 30 μg/L	ELISA	344 (AG: 148)	58.8 ± 14.2	31.8 (24.4–39.9)	98.0 (94.9–99.4)	0.629 (0.565–0.692
			PG I < 43.6 μg/L				37.8 (30.0–46.2)	95.9 (92.1–98.2)	
			PGI/PGII ratio < 3				30.6 (23.3–38.7)	97.4 (94.1–99.2)	0.679 (0.619–0.738)
			PGI/PGII ratio < 7				50.3 (42.0–58.7)	83.7 (77.7–88.6)	
Whary [44] (2020)	Single center, Colombia	AG/GC	PGI/PGII ratio n/a value for AG/GC	ELISA	153	n/a	44.7	83	n/a
Miftahussurur [45] (2020)	Multicenter, Southeast Asia	AG, *Helicobacter pylori* infection	PG I ≤ 70 ng/mL and PGI/PGII ratio ≤ 3	ELISA	1206	44 (13–88)	15.9	96.9	n/a
			PGII ≥ 10.35 ng/mL				72.6	56.9	0.664
			PGI/PGII ratio ≤ 4.95				66.2	67.5	0.718
Zeng [35] 2020	Single center, China	AG, GC	PG I < 71.56 μg/L	ELISA	197 (GC: 86 AG: 61)	n/a	77.1	66.0	0.719 (0.621–0.816)
			PG I/II ratio < 5.6				60.1	82.0	0.755 (0.666–0.844)
			PG I < 71.56 μg/L, PG I/II ratio < 5.6				67.2	84.0	0.807 (0.727–0.888)
Mattar [46] (2020)	Single center, Brasil	AG	PGI < 30 µg/L	n/a	308 (corpus AG: 29)	64.6 ± 10.3	50 (27.8–72.1)	93.2 (84.3–97.5)	n/a
			PGI < 30 µg/L		308 (multifocal AG: 29)		42.1 (21.1–66)	n/a	n/a
			PGI/PGII ratio < 3		308 (corpus AG: 29)		55 (32–76.2)	93.2 (84.3–97.4%)	n/a
			PGI/PGII ratio < 3		308 (multifocal AG: 29)		21 (6.9–46)	n/a	n/a
Wang [47] (2020)	Single center, China	AG, GC	PG I < 91.45 μg/L	ELISA	630 (AG: 245)	55.2 ± 10.8	73.15	50.00	0.691 (0.652–0.876)
			PGI/PGII ratio < 9				72.70	53.31	0.650 (0.612–0.856)
Mezmale [48] (2019)	Multicenter, Kazakhstan	AG	PG I ≤ 70 ng/mL and PGI/PGII ratio ≤ 3	L-AA	157	51 ± 6.9	50.0 (1.2–98.7)	50.0 (1.2–98.7)	n/a
			PG I ≤ 30 ng/mL and PGI/PGII ratio ≤ 2				73.5 (65.8–80.3)	90.9 (85.3–94.9)	n/a
Loong [37] (2017)	Single center, Malaysia	AG, IM	PGI ≤ 87.2 μg/L)	ELISA	69 (AG: 34 AG)	56.2 ± 16	66.7	85.3	0.659
			PG I/II ratio ≤ 10				83.3	77.9	0.902
			G-17b < 5.6 pmol/L				68.8	44.8	<0.5
Leja [36] (2017)	Case-control Multicenter, Latvia	corpus AG	PGI/PGII < 6.9	ELISA Biohit	805 (AG: 50)	51 (18–88)	70.0 (57.3–82.7)	62.6 (59.2–66.1)	0.7
			PGI/PGII < 4.1	ELISA Vector Best			70.0 (57.3–82.7)	71.5 (68.3–74.7)	0.76
			PGI/PGII < 2.7	L-AA			70.0 (57.3–82.7)	71.9 (68.7–75.1)	0.77
Bang [38] (2019)	Meta-analysis: 14 studies for AG, 43 for GC	AG, GC	PG I ≤ 70 ng/mL and PGI/PGII ratio ≤ 3	ELISA, L-TIA, RIA	5541 (AG: 2220)	n/a	59 (38–78)	89 (70–97)	0.81 (0.77–0.84
Huang [49] (2015)	Meta-analysis: 14 studies for AG, 17 for GC	AG, GC	PGI/PGII ratio ≤ 3	ELISA, L-TIA, RIA, CLIA	AG: 2220	n/a	50 (28–72)	94 (82–98)	0.85 (0.81–0.88)

CI, confidence interval; n/a, not available; AG, atrophic gastritis; IM, intestinal metaplasia; GC, gastric cancer; HpAb, antibodies to *H. pylori* [EIU]; EIU, enzyme immune units; PGI, pepsinogen I; PGII, pepsinogen II; G-17b, basal; Gastrin-17 [pmol/L]; L-AA, latex-agglutination assay; ELISA, enzyme-linked immunosorbent assay; CMIA, Chemiluminescent Microparticle Immuno Assay; CLEIA, ChemiLuminescent Enzyme ImmunoAssay; RIA, Radio Immuno Assay; L-TIA, Latex turbidimetric immunoassa.

## References

[1] Bray F., Laversanne M., Sung H., Ferlay J., Siegel R.L., Soerjomataram I., Jemal A. (2024). Global Cancer Statistics 2022: GLOBOCAN Estimates of Incidence and Mortality Worldwide for 36 Cancers in 185 Countries. CA Cancer J. Clin..

[2] Laurén P. (1965). The Two Histological Main Types of Gastric Carcinoma: Diffuse and so-Called Intestinal-Type Carcinoma. Acta Pathol. Microbiol. Scand..

[3] Sung H., Ferlay J., Siegel R.L., Laversanne M., Soerjomataram I., Jemal A., Bray F. (2021). Global Cancer Statistics 2020: GLOBOCAN Estimates of Incidence and Mortality Worldwide for 36 Cancers in 185 Countries. CA Cancer J. Clin..

[4] Correa P. (1988). A Human Model of Gastric Carcinogenesis. Cancer Res..

[5] Leung W.K., Lin S.R., Ching J.Y.L., To K.F., Ng E.K.W., Chan F.K.L., Lau J.Y.W., Sung J.J.Y. (2004). Factors Predicting Progression of Gastric Intestinal Metaplasia: Results of a Randomised Trial on *Helicobacter pylori* Eradication. Gut.

[6] Fukase K., Kato M., Kikuchi S., Inoue K., Uemura N., Okamoto S., Terao S., Amagai K., Hayashi S., Asaka M. (2008). Effect of Eradication of *Helicobacter pylori* on Incidence of Metachronous Gastric Carcinoma after Endoscopic Resection of Early Gastric Cancer: An Open-Label, Randomised Controlled Trial. Lancet.

[7] Massironi S., Zilli A., Elvevi A., Invernizzi P. (2019). The Changing Face of Chronic Autoimmune Atrophic Gastritis: An Updated Comprehensive Perspective. Autoimmun. Rev..

[8] Coati I., Fassan M., Farinati F., Graham D.Y., Genta R.M., Rugge M. (2015). Autoimmune Gastritis: Pathologist’s Viewpoint. World J. Gastroenterol..

[9] Capelle L.G., de Vries A.C., Haringsma J., Ter Borg F., de Vries R.A., Bruno M.J., van Dekken H., Meijer J., van Grieken N.C.T., Kuipers E.J. (2010). The Staging of Gastritis with the OLGA System by Using Intestinal Metaplasia as an Accurate Alternative for Atrophic Gastritis. Gastrointest. Endosc..

[10] de Vries A.C., van Grieken N.C.T., Looman C.W.N., Casparie M.K., de Vries E., Meijer G.A., Kuipers E.J. (2008). Gastric Cancer Risk in Patients with Premalignant Gastric Lesions: A Nationwide Cohort Study in the Netherlands. Gastroenterology.

[11] Den Hollander W.J., Holster I.L., Den Hoed C.M., Capelle L.G., Tang T.J., Anten M.P., Prytz-Berset I., Witteman E.M., Ter Borg F., Hartog G.D. (2019). Surveillance of Premalignant Gastric Lesions: A Multicentre Prospective Cohort Study from Low Incidence Regions. Gut.

[12] Pimentel-Nunes P., Libânio D., Marcos-Pinto R., Areia M., Leja M., Esposito G., Garrido M., Kikuste I., Megraud F., Matysiak-Budnik T. (2019). Management of Epithelial Precancerous Conditions and Lesions in the Stomach (MAPS II): European Society of Gastrointestinal Endoscopy (ESGE), European Helicobacter and Microbiota Study Group (EHMSG), European Society of Pathology (ESP), and Sociedade Port. Endoscopy.

[13] Leja M., Park J.Y., Murillo R., Liepniece-Karele I., Isajevs S., Kikuste I., Rudzite D., Krike P., Parshutin S., Polaka I. (2017). Multicentric Randomised Study of *Helicobacter pylori* Eradication and Pepsinogen Testing for Prevention of Gastric Cancer Mortality: The GISTAR Study. BMJ Open.

[14] Whiting J.L., Sigurdsson A., Rowlands D.C., Hallissey M.T., Fielding J.W.L. (2002). The Long Term Results of Endoscopic Surveillance of Premalignant Gastric Lesions. Gut.

[15] Areia M., Spaander M.C.W., Kuipers E.J., Dinis-Ribeiro M. (2018). Endoscopic Screening for Gastric Cancer: A Cost-Utility Analysis for Countries with an Intermediate Gastric Cancer Risk. United Eur. Gastroenterol. J..

[16] Romańczyk M., Ostrowski B., Budzyń K., Koziej M., Wdowiak M., Romańczyk T., Błaszczyńska M., Kajor M., Januszewski K., Zajęcki W. (2022). The Role of Endoscopic and Demographic Features in the Diagnosis of Gastric Precancerous Conditions. Pol. Arch. Intern. Med..

[17] Honing J., Tan W., Dieninyte E., O’Donovan M., Brosens L., Weusten B., di Pietro M. (2023). Adequacy of Endoscopic Recognition and Surveillance of Gastric Intestinal Metaplasia and Atrophic Gastritis: A Multicentre Retrospective Study in Low Incidence Countries. PLoS ONE.

[18] Miwata T., Quach D.T., Hiyama T., Aoki R., Le H.M., Tran P.L.N., Ito M., Tanaka S., Arihiro K., Uemura N. (2015). Interobserver and Intraobserver Agreement for Gastric Mucosa Atrophy. BMC Gastroenterol..

[19] Kaji K., Hashiba A., Uotani C., Yamaguchi Y., Ueno T., Ohno K., Takabatake I., Wakabayashi T., Doyama H., Ninomiya I. (2018). Grading of Atrophic Gastritis Is Useful for Risk Stratification in Endoscopic Screening for Gastric Cancer. Am. J. Gastroenterol..

[20] Shichijo S., Hirata Y., Niikura R., Hayakawa Y., Yamada A., Ushiku T., Fukayama M., Koike K. (2016). Histologic Intestinal Metaplasia and Endoscopic Atrophy Are Predictors of Gastric Cancer Development after *Helicobacter pylori* Eradication. Gastrointest. Endosc..

[21] Kimura K., Takemoto T. (1969). An Endoscopic Recognition of the Atrophic Border and Its Significance in Chronic Gastritis. Endoscopy.

[22] Rokkas T., Ekmektzoglou K. (2023). Current Role of Narrow Band Imaging in Diagnosing Gastric Intestinal Metaplasia: A Systematic Review and Meta-Analysis of Its Diagnostic Accuracy. Ann. Gastroenterol..

[23] Esposito G., Pimentel-Nunes P., Angeletti S., Castro R., Libânio D., Galli G., Lahner E., Di Giulio E., Annibale B., Dinis-Ribeiro M. (2019). Endoscopic Grading of Gastric Intestinal Metaplasia (EGGIM): A Multicenter Validation Study. Endoscopy.

[24] Marcos P., Brito-Gonçalves G., Libânio D., Pita I., Castro R., Sá I., Dinis-Ribeiro M., Pimentel-Nunes P. (2020). Endoscopic Grading of Gastric Intestinal Metaplasia on Risk Assessment for Early Gastric Neoplasia: Can We Replace Histology Assessment Also in the West?. Gut.

[25] Kanemitsu T., Uedo N., Ono T., Nimura S., Hasegawa R., Imamura K., Ohtsu K., Ono Y., Miyaoka M., Ueki T. (2023). Magnifying Endoscopy with Narrow-band Imaging for Diagnosis of Subtype of Gastric Intestinal Metaplasia. J. Gastroenterol. Hepatol..

[26] Sieg A., Hachmoeller-Eisenbach U., Eisenbach T. (2001). Prospective Evaluation of Complications in Outpatient GI Endoscopy: A Survey among German Gastroenterologists. Gastrointest. Endosc..

[27] Romańczyk M., Ostrowski B., Barański K., Romańczyk T., Błaszczyńska M., Budzyń K., Didkowska J., Wojciechowska U., Hartleb M. (2023). Potential Benefits of One-Time Gastroscopy in Searching for Precancerous Conditions. Pol. Arch. Intern. Med..

[28] Budzyń K., Pelczar M., Romańczyk M., Barański K., Hartleb M. (2024). The Survey of Willingness to Undergo Screening Gastroscopy in the Population of Low-to-Moderate Prevalence Rate of Esophageal and Gastric Cancers. Pol. Arch. Intern. Med..

[29] Koivurova O.P., Koskela R., Blomster T., Ala-Rämi A., Lumme H., Kettunen O., Hukkanen J., Karttunen T.J., Mäkinen M., Ronkainen J. (2021). Serological Biomarker Panel in Diagnosis of Atrophic Gastritis and *Helicobacter pylori* Infection in Gastroscopy Referral Patients: Clinical Validation of the New-Generation Gastropanel^®^ Test. Anticancer Res..

[30] Chapelle N., Martin J., Osmola M., Hémont C., Leroy M., Vibet M.A., Tougeron D., Moussata D., Lamarque D., Bigot-Corbel E. (2023). Serum Pepsinogens Can Help to Discriminate between H. Pylori-Induced and Auto-Immune Atrophic Gastritis: Results from a Prospective Multicenter Study. Dig. Liver Dis..

[31] Chapelle N., Osmola M., Martin J., Blin J., Leroy M., Jirka I., Moussata D., Lamarque D., Olivier R., Tougeron D. (2022). Serum Pepsinogens Combined with New Biomarkers Testing Using Chemiluminescent Enzyme Immunoassay for Non-Invasive Diagnosis of Atrophic Gastritis: A Prospective, Multicenter Study. Diagnostics.

[32] Nguyen C.L., Dao T.T., Phi T.T.N., Nguyen T.P., Pham V.T., Vu T.K. (2022). Serum Pepsinogen: A Potential Non-Invasive Screening Method for Moderate and Severe Atrophic Gastritis among an Asian Population. Ann. Med. Surg..

[33] Ogutmen Koc D., Bektas S. (2021). Serum Pepsinogen Levels and OLGA/OLGIM Staging in the Assessment of Atrophic Gastritis Types. Postgrad. Med. J..

[34] Cai H.L., Tong Y.L. (2021). Association of Serum Pepsinogen with Degree of Gastric Mucosal Atrophy in an Asymptomatic Population. World J. Clin. Cases.

[35] Zeng W., Zhang S., Yang L., Wei W., Gao J., Guo N., Wu F. (2020). Serum MiR-101-3p Combined with Pepsinogen Contributes to the Early Diagnosis of Gastric Cancer. BMC Med. Genet..

[36] Leja M., Camargo M.C., Polaka I., Isajevs S., Liepniece-Karele I., Janciauskas D., Rudzite D., Kikuste I., Vanags A., Kojalo I. (2017). Detection of Gastric Atrophy by Circulating Pepsinogens: A Comparison of Three Assays. Helicobacter.

[37] Loong T.H., Soon N.C., Nik Mahmud N.R.K., Naidu J., Abdul Rani R., Abdul Hamid N., Hikmah Elias M., Rose I.M., Tamil A., Mokhtar N.M. (2017). Serum Pepsinogen and Gastrin-17 as Potential Biomarkers for Pre-malignant Lesions in the Gastric Corpus. Biomed. Rep..

[38] Bang C.S., Lee J.J., Baik G.H. (2019). Prediction of Chronic Atrophic Gastritis and Gastric Neoplasms by Serum Pepsinogen Assay: A Systematic Review and Meta-Analysis of Diagnostic Test Accuracy. J. Clin. Med..

[39] Lin X.K., Wang W.L. (2023). Analysis of High Risk Factors for Chronic Atrophic Gastritis. Saudi J. Gastroenterol..

[40] Reza Sivandzadeh G., Amiri Zadeh Fard S., Zahmatkesh A., Hossein Anbardar M., Lankarani K.B., Author C. (2023). Value of Serological Biomarker Panel in Diagnosis of Atrophic Gastritis and *Helicobacter pylori* Infection. Middle East. J. Dig. Dis..

[41] Huang R.J., Park S., Shen J., Longacre T., Ji H., Hwang J.H. (2022). Pepsinogens and Gastrin Demonstrate Low Discrimination for Gastric Precancerous Lesions in a Multi-Ethnic United States Cohort. Clin. Gastroenterol. Hepatol..

[42] Miftahussurur M., Waskito L.A., Syam A.F., Nusi I.A., Dewa Nyoman Wibawa I., Rezkitha Y.A.A., Fauzia K.A., Siregar G.A., Akil F., Waleleng B.J. (2022). Serum Pepsinogen Level as a Biomarker for Atrophy, Reflux Esophagitis, and Gastric Cancer Screening in Indonesia. J. Res. Med. Sci..

[43] Chapelle N., Petryszyn P., Blin J., Leroy M., Le Berre-Scoul C., Jirka I., Neunlist M., Moussata D., Lamarque D., Olivier R. (2020). A Panel of Stomach-Specific Biomarkers (GastroPanel^®^) for the Diagnosis of Atrophic Gastritis: A Prospective, Multicenter Study in a Low Gastric Cancer Incidence Area. Helicobacter.

[44] Whary M.T., Avenia J.M.R., Bravo L.E., Lofgren J.L., Lertpiriyapong K., Mera-Giler R., Piazuelo M.B., Correa P., Peek R.M., Wilson K.T. (2020). Contrasting Serum Biomarker Profiles in Two Colombian Populations with Different Risks for Progression of Premalignant Gastric Lesions during Chronic *Helicobacter pylori* Infection. Cancer Epidemiol..

[45] Miftahussurur M., Agung Waskito L., Aftab H., Vilaichone R., Subsomwong P., Nusi I.A., Syam A.F., Ratanachu-Ek T., Doohan D., Siregar G. (2020). Serum Pepsinogens as a Gastric Cancer and Gastritis Biomarker in South and Southeast Asian Populations. PLoS ONE.

[46] Mattar R., Marques S.B., Ribeiro I.B., Visconti T.A.d.C., Funari M., de Moura E.G.H. (2020). Diagnostic Accuracy of Gastropanel^®^ for Atrophic Gastritis in Brazilian Subjects and the Effect of Proton Pump Inhibitors. Arq. Gastroenterol..

[47] Wang Y., Liu X., Wang L., Zhang Z., Li Z., Li M. (2020). A Comparative Study on Changes in Intestinal Flora, Pepsinogen and Gastrin in Patients with Gastric Cancer and Atrophic Gastritis. JBUON.

[48] Mezmale L., Isajevs S., Bogdanova I., Polaka1 I., Krigere A., Rudzite D., Rudule A., Kikuste I., Parshutin S., Tazhedinov A. (2019). Prevalence of Atrophic Gastritis in Kazakhstan and the Accuracy of Pepsinogen Tests to Detect Gastric Mucosal Atrophy. Asian Pac. J. Cancer Prev..

[49] Huang Y.K., Yu J.C., Kang W.M., Ma Z.Q., Ye X., Tian S.B., Yan C. (2015). Significance of Serum Pepsinogens as a Biomarker for Gastric Cancer and Atrophic Gastritis Screening: A Systematic Review and Meta-Analysis. PLoS ONE.

[50] Dinis-Ribeiro M., Areia M., De Vries A.C., Marcos-Pinto R., Monteiro-Soares M., Oconnor A., Pereira C., Pimentel-Nunes P., Correia R., Ensari A. (2012). Management of Precancerous Conditions and Lesions in the Stomach (MAPS): Guideline from the European Society of Gastrointestinal Endoscopy (ESGE), European Helicobacter Study Group (EHSG), European Society of Pathology (ESP), and the Sociedade Portuguesa de Endoscopia Digestiva (SPED). Endoscopy.

[51] Malfertheiner P., Megraud F., Rokkas T., Gisbert J.P., Liou J.M., Schulz C., Gasbarrini A., Hunt R.H., Leja M., O’Morain C. (2022). Management of *Helicobacter pylori* Infection: The Maastricht VI/Florence Consensus Report. Gut.

[52] Sugano K., Tack J., Kuipers E.J., Graham D.Y., El-Omar E.M., Miura S., Haruma K., Asaka M., Uemura N., Malfertheiner P. (2015). Kyoto Global Consensus Report on *Helicobacter pylori* Gastritis. Gut.

[53] Kamada T., Maruyama Y., Monobe Y., Haruma K. (2022). Endoscopic Features and Clinical Importance of Autoimmune Gastritis. Dig. Endosc..

[54] Kang D., Lim C.-H., Kim J.S., Cho Y.K., Park J.M., Choi M.-G. (2023). Impact of Autoimmune Gastritis on Occurrence of Metachronous Gastric Neoplasms after Endoscopic Resection for Gastric Neoplasms. Cancers.

[55] Watson S.A., Grabowska A.M., El-Zaatari M., Takhar A. (2006). Gastrin—Active Participant or Bystander in Gastric Carcinogenesis?. Nat. Rev. Cancer.

[56] Copps J., Murphy R.F., Lovas S. (2009). The Production and Role of Gastrin-17 and Gastrin-17-Gly in Gastrointestinal Cancers. Protein Pept. Lett..

[57] Leja M., Kupcinskas L., Funka K., Sudraba A., Jonaitis L., Ivanauskas A., Janciauskas D., Kuidelis G., Chiu H.M., Lin J.T. (2011). Value of Gastrin-17 in Detecting Antral Atrophy. Adv. Med. Sci..

[58] Korstanje A., Den Hartog G., Biemond I., Lamers C.B.H.W. (2002). The Serological Gastric Biopsy: A Non-Endoscopical Diagnostic Approach in Management of the Dyspeptic Patient Signi Cance for Primary Care Based on a Survey of the Literature. Scand. J. Gastroenterol. Suppl..

[59] Lundell L., Vieth M., Gibson F., Nagy P., Kahrilas P.J. (2015). Systematic Review: The Effects of Long-Term Proton Pump Inhibitor Use on Serum Gastrin Levels and Gastric Histology. Aliment. Pharmacol. Ther..

[60] Zagari R.M., Rabitti S., Greenwood D.C., Eusebi L.H., Vestito A., Bazzoli F. (2017). Systematic Review with Meta-analysis: Diagnostic Performance of the Combination of Pepsinogen, Gastrin-17 and Anti-*Helicobacter pylori* Antibodies Serum Assays for the Diagnosis of Atrophic Gastritis. Aliment. Pharmacol. Ther..

[61] Syrjänen K., Eskelinen M., Peetsalu A., Sillakivi T., Sipponen P., Härkönen M., Paloheimo L., Mäki M., Tiusanen T., Suovaniemi O. (2019). GastroPanel^®^ Biomarker Assay: The Most Comprehensive Test for *Helicobacter pylori* Infection and Its Clinical Sequelae. A Critical Review. Anticancer Res..

[62] Agréus L., Kuipers E.J., Kupcinskas L., Malfertheiner P., Di Mario F., Leja M., Mahachai V., Yaron N., van Oijen M., Perez Perez G. (2012). Rationale in Diagnosis and Screening of Atrophic Gastritis with Stomach-Specific Plasma Biomarkers. Scand. J. Gastroenterol..

[63] Mäki M., Söderström D., Paloheimo L., Hendolin P., Suovaniemi O., Syrjänen K. (2020). *Helicobacter pylori* (Hp) IgG ELISA of the New-Generation GastroPanel^®^ Is Highly Accurate in Diagnosis of Hp-Infection in Gastroscopy Referral Patients. Anticancer Res..

[64] Syrjänen K. (2022). Accuracy of Serum Biomarker Panel (GastroPanel ^®^) in the Diagnosis of Atrophic Gastritis of the Corpus. Systematic Review and Meta-Analysis. Anticancer Res..

[65] Chiang T., Maeda M., Yamada H., Chan C., Chen S.L., Chiu S.Y., Chen Y., Chou Y., Shieh C., Liu C. (2021). Risk Stratification for Gastric Cancer after *Helicobacter pylori* Eradication: A Population-based Study on Matsu Islands. J. Gastroenterol. Hepatol..

[66] Chiang T.H., Chen Y.N., Chen Y.R., Tseng Y.H., Shieh C.F., Liu C.Y., Chiu H.M., Chiang H., Shun C.T., Wu M.S. (2022). Brand Interchangeability of Pepsinogen Tests in the Real-World Setting after Eradication of *Helicobacter pylori*: A Community-Based Study. BMC Gastroenterol..

[67] Syrjänen K. (2016). A Panel of Serum Biomarkers (Gastropanel^®^) in Non-Invasive Diagnosis of Atrophic Gastritis. Systematic Review and Meta-Analysis. Anticancer Res..

[68] McNicholl A.G., Forné M., Barrio J., De la Coba C., González B., Rivera R., Esteve M., Fernandez-Bañares F., Madrigal B., Gras-Miralles B. (2014). Accuracy of GastroPanel for the Diagnosis of Atrophic Gastritis. Eur. J. Gastroenterol. Hepatol..

[69] Grad C., Pop A., Gaborean E., Grad S., Dumitrascu D. (2021). Value of GastroPanel in the Diagnosis of Atrophic Gastritis. Exp. Ther. Med..

[70] Franceschi M., Rodriguez-Castro K.I., Ferronato A., Massella A., Brozzi L., Panozzo M.P., Antico A., Pertoldi B., Morini A., Barchi A. (2022). A Non-Invasive Combined Strategy to Improve the Appropriateness of Upper Gastrointestinal Endoscopy. Acta Biomed..

[71] Pei B., Wen Z., Yang Q., Wang J., Cao Q., Dai L., Li X. (2022). Risk Factors Analysis and Prediction Model Establishment of Intestinal Metaplasia or Dysplasia in Patients with Chronic Atrophic Gastritis: A Multi-Center Retrospective Study. Front. Med..

[72] Guo X., Schreurs M.W.J., Marijnissen F.E., Mommersteeg M.C., Nieuwenburg S.A.V., Doukas M., Erler N.S., Capelle L.G., Bruno M.J., Peppelenbosch M.P. (2023). Increased Prevalence of Autoimmune Gastritis in Patients with a Gastric Precancerous Lesion. J. Clin. Med..

[73] Dondov G., Amarbayasgalan D., Batsaikhan B., Badamjav T., Batbaatar B., Tuvdenjamts B., Tumurbat N., Davaa B., Purevdorj E., Nyamaa B. (2022). Diagnostic Performances of Pepsinogens and Gastrin-17 for Atrophic Gastritis and Gastric Cancer in Mongolian Subjects. PLoS ONE.

[74] Ambros V. (2004). The Functions of Animal MicroRNAs. Nature.

[75] Gupta R.A., Shah N., Wang K.C., Kim J., Horlings H.M., Wong D.J., Tsai M.-C., Hung T., Argani P., Rinn J.L. (2010). Long Non-Coding RNA HOTAIR Reprograms Chromatin State to Promote Cancer Metastasis. Nature.

[76] Link J., Thon C., Petkevicius V., Steponaitiene R., Malfertheiner P., Kupcinskas J., Link A. (2023). The Translational Impact of Plant-Derived Xeno-MiRNA MiR-168 in Gastrointestinal Cancers and Preneoplastic Conditions. Diagnostics.

[77] Hansen T.B., Jensen T.I., Clausen B.H., Bramsen J.B., Finsen B., Damgaard C.K., Kjems J. (2013). Natural RNA Circles Function as Efficient MicroRNA Sponges. Nature.

[78] Meltzer P.S. (2005). Small RNAs with Big Impacts. Nature.

[79] Leja M., Wex T., Malfertheiner P. (2012). Markers for Gastric Cancer Premalignant Lesions: Where Do We Go?. Dig. Dis..

[80] Link A., Kupcinskas J., Wex T., Malfertheiner P. (2012). Macro-Role of MicroRNA in Gastric Cancer. Dig. Dis..

[81] Link A., Schirrmeister W., Langner C., Varbanova M., Bornschein J., Wex T., Malfertheiner P. (2015). Differential Expression of MicroRNAs in Preneoplastic Gastric Mucosa. Sci. Rep..

[82] Cortés-Márquez A.C., Mendoza-Elizalde S., Arenas-Huertero F., Trillo-Tinoco J., Valencia-Mayoral P., Consuelo-Sánchez A., Zarate-Franco J., Dionicio-Avendaño A.R., Herrera-Esquivel J.d.J., Recinos-Carrera E.G. (2018). Differential Expression of MiRNA-146a and MiRNA-155 in Gastritis Induced by *Helicobacter pylori* Infection in Paediatric Patients, Adults, and an Animal Model. BMC Infect. Dis..

[83] Vasapolli R., Venerito M., Schirrmeister W., Thon C., Weigt J., Wex T., Malfertheiner P., Link A. (2021). Inflammatory MicroRNAs in Gastric Mucosa Are Modulated by *Helicobacter pylori* Infection and Proton-Pump Inhibitors but Not by Aspirin or NSAIDs. PLoS ONE.

[84] Matsushima K., Isomoto H., Inoue N., Nakayama T., Hayashi T., Nakayama M., Nakao K., Hirayama T., Kohno S. (2011). MicroRNA Signatures in *Helicobacter pylori* Infected Gastric Mucosa. Int. J. Cancer.

[85] Jonaitis P., Kupcinskas L., Kupcinskas J. (2021). Molecular Alterations in Gastric Intestinal Metaplasia. Int. J. Mol. Sci..

[86] Okubo M., Tahara T., Shibata T., Yamashita H., Nakamura M., Yoshioka D., Yonemura J., Kamiya Y., Ishizuka T., Nakagawa Y. (2010). Association between Common Genetic Variants in Pre-MicroRNAs and the Clinicopathological Characteristics and Survival of Gastric Cancer Patients. Exp. Ther. Med..

[87] Christodoulidis G., Koumarelas K.-E., Kouliou M.-N., Thodou E., Samara M. (2024). Gastric Cancer in the Era of Epigenetics. Int. J. Mol. Sci..

[88] Prinz C., Mese K., Weber D. (2021). MicroRNA Changes in Gastric Carcinogenesis: Differential Dysregulation during *Helicobacter pylori* and EBV Infection. Genes.

[89] Saito M., Kono K. (2021). Landscape of EBV-Positive Gastric Cancer. Gastric Cancer.

[90] Petkevicius V., Thon C., Steponaitiene R., Skieceviciene J., Janciauskas D., Jechorek D., Malfertheiner P., Kupcinskas J., Link A. (2022). Differential Expression of Long Noncoding RNA HOTAIR in Intestinal Metaplasia and Gastric Cancer. Clin. Transl. Gastroenterol..

[91] Link A., Kupcinskas J. (2018). MicroRNAs as Non-Invasive Diagnostic Biomarkers for Gastric Cancer: Current Insights and Future Perspectives. World J. Gastroenterol..

[92] Liu H., Li P.-W., Yang W.-Q., Mi H., Pan J.-L., Huang Y.-C., Hou Z.-K., Hou Q.-K., Luo Q., Liu F.-B. (2019). Identification of Non-Invasive Biomarkers for Chronic Atrophic Gastritis from Serum Exosomal MicroRNAs. BMC Cancer.

[93] Bado A., Levasseur S., Attoub S., Kermorgant S., Laigneau J.-P., Bortoluzzi M.-N., Moizo L., Lehy T., Guerre-Millo M., Le Marchand-Brustel Y. (1998). The Stomach Is a Source of Leptin. Nature.

[94] Inagaki-Ohara K. (2019). Gastric Leptin and Tumorigenesis: Beyond Obesity. Int. J. Mol. Sci..

[95] Boutari C., Perakakis N., Mantzoros C.S. (2018). Association of Adipokines with Development and Progression of Nonalcoholic Fatty Liver Disease. Endocrinol. Metab..

[96] Arita S., Inagaki-Ohara K. (2019). High-Fat-Diet–Induced Modulations of Leptin Signaling and Gastric Microbiota Drive Precancerous Lesions in the Stomach. Nutrition.

[97] Francois F., Roper J., Goodman A.J., Pei Z., Ghumman M., Mourad M., de Perez A.Z.O., Perez-Perez G.I., Tseng C.-H., Blaser M.J. (2007). The Association of Gastric Leptin with Oesophageal Inflammation and Metaplasia. Gut.

[98] Capelle L.G., De Vries A.C., Haringsma J., Steyerberg E.W., Looman C.W.N., Nagtzaam N.M.A., Van Dekken H., Ter Borg F., De Vries R.A., Kuipers E.J. (2009). Serum Levels of Leptin As Marker For Patients At High Risk of Gastric Cancer. Helicobacter.

[99] Kendall B.J., Macdonald G.A., Hayward N.K., Prins J.B., Brown I., Walker N., Pandeya N., Green A.C., Webb P.M., Whiteman D.C. (2007). Leptin and the Risk of Barrett’s Oesophagus. Gut.

[100] Zhao L. (2005). Possible Involvement of Leptin and Leptin Receptor in Developing Gastric Adenocarcinoma. World J. Gastroenterol..

[101] Zhao X., Huang K., Zhu Z., Chen S., Hu R. (2007). Correlation between Expression of Leptin and Clinicopathological Features and Prognosis in Patients with Gastric Cancer. J. Gastroenterol. Hepatol..

[102] Ishikawa M. (2006). Expression Pattern of Leptin and Leptin Receptor (OB-R) in Human Gastric Cancer. World J. Gastroenterol..

[103] Monks M., Irakleidis F., Tan Peng H. (2019). Complex Interaction of Adiponectin-Mediated Pathways on Cancer Treatment: A Novel Therapeutic Target. J. Cancer Metastasis Treat..

[104] Ando T., Ishikawa T., Takagi T., Imamoto E., Kishimoto E., Okajima A., Uchiyama K., Handa O., Yagi N., Kokura S. (2013). Impact of *Helicobacter pylori* Eradication on Circulating Adiponectin in Humans. Helicobacter.

[105] Torisu T., Takata Y., Ansai T., Matsumoto T., Sonoki K., Soh I., Awano S., Yoshida A., Hamasaki T., Kagiyama S. (2009). Possible Association of Atrophic Gastritis and Arterial Stiffness in Healthy Middle-Aged Japanese. J. Atheroscler. Thromb..

[106] Kishida K., Funahashi T., Shimomura I. (2014). Adiponectin as a Routine Clinical Biomarker. Best. Pract. Res. Clin. Endocrinol. Metab..

[107] Ishikawa M., Kitayama J., Kazama S., Hiramatsu T., Hatano K., Nagawa H. (2005). Plasma Adiponectin and Gastric Cancer. Clin. Cancer Res..

[108] Seker M., Bilici A., Sonmez B., Ustaalioğlu B.B.O., Gumus M., Gozu H., Sargin M., Orcun A., Gezen C., Eser M. (2010). The Association of Serum Adiponectin Levels with Histopathological Variables in Gastric Cancer Patients. Med. Oncol..

[109] Laron Z. (2001). Insulin-like Growth Factor 1 (IGF-1): A Growth Hormone. Mol. Pathol..

[110] Werner H., Bruchim I. (2009). The Insulin-like Growth Factor-I Receptor as an Oncogene. Arch. Physiol. Biochem..

[111] Yi H.K., Hwang P.H., Yang D.-H., Kang C.-W., Lee D.-Y. (2001). Expression of the Insulin-like Growth Factors (IGFs) and the IGF-Binding Proteins (IGFBPs) in Human Gastric Cancer Cells. Eur. J. Cancer.

[112] Kędzierska L., Madej-Michniewicz A., Marczuk N., Dołęgowska B., Starzyńska T., Błogowski W. (2020). Clinical Significance of Various Growth Factors in Patients with Different Gastric Neoplasms. Am. J. Transl. Res..

[113] Ennishi D., Shitara K., Ito H., Hosono S., Watanabe M., Ito S., Sawaki A., Yatabe Y., Yamao K., Tajima K. (2011). Association between Insulin-like Growth Factor-1 Polymorphisms and Stomach Cancer Risk in a Japanese Population. Cancer Sci..

[114] Pavelić K., Kolak T., Kapitanović S., Radošević S., Spaventi Š., Krušlin B., Pavelić J. (2003). Gastric Cancer: The Role of Insulin-like Growth Factor 2 (IGF 2) and Its Receptors (IGF 1R and M6-P/IGF 2R). J. Pathol..

[115] Ustundag Y., Sahin H., İlikhan S., Dogan B.G., Kokturk F., Kar F. (2013). *Helicobacter pylori* Eradication Does Not Change Circulating Insulin-Like Growth Factor 1 and Insulin-Like Growth Factor Binding Protein 3 Levels in Patients with and without Precancerous Gastric Lesions. Am. J. Med. Sci..

[116] Arnold M., Park J.Y., Camargo M.C., Lunet N., Forman D., Soerjomataram I. (2020). Is Gastric Cancer Becoming a Rare Disease? A Global Assessment of Predicted Incidence Trends to 2035. Gut.

[117] Anderson W.F., Rabkin C.S., Turner N., Fraumeni J.F., Rosenberg P.S., Camargo M.C. (2018). The Changing Face of Noncardia Gastric Cancer Incidence Among US Non-Hispanic Whites. JNCI J. Natl. Cancer Inst..

[118] Blaser M.J., Chen Y. (2018). A New Gastric Cancer Among Us. JNCI J. Natl. Cancer Inst..

[119] Song M., Camargo M.C., Katki H.A., Weinstein S.J., Männistö S., Albanes D., Surcel H.-M., Rabkin C.S. (2022). Association of Antiparietal Cell and Anti-Intrinsic Factor Antibodies with Risk of Gastric Cancer. JAMA Oncol..

[120] Song M., Latorre G., Ivanovic-Zuvic D., Camargo M.C., Rabkin C.S. (2019). Autoimmune Diseases and Gastric Cancer Risk: A Systematic Review and Meta-Analysis. Cancer Res. Treat..

[121] Zádori N., Szakó L., Váncsa S., Vörhendi N., Oštarijaš E., Kiss S., Frim L., Hegyi P., Czimmer J. (2021). Six Autoimmune Disorders Are Associated with Increased Incidence of Gastric Cancer: A Systematic Review and Meta-Analysis of Half a Million Patients. Front. Immunol..

[122] Landgren A.M., Landgren O., Gridley G., Dores G.M., Linet M.S., Morton L.M. (2011). Autoimmune Disease and Subsequent Risk of Developing Alimentary Tract Cancers among 4.5 Million US Male Veterans. Cancer.

[123] Osmola M., Hemont C., Chapelle N., Vibet M.-A., Tougeron D., Moussata D., Lamarque D., Bigot-Corbel E., Masson D., Blin J. (2023). Atrophic Gastritis and Autoimmunity: Results from a Prospective, Multicenter Study. Diagnostics.

[124] Presotto F., Sabini B., Cecchetto A., Plebani M., Lazzari F.D., Pedini B., Betterle C. (2003). *Helicobacter pylori* Infection and Gastric Autoimmune Diseases: Is There a Link?. Helicobacter.

[125] Allakky A. (2023). Exploring the Association of *Helicobacter pylori* with Anti-Intrinsic Factor and Anti-Parietal Cell Antibodies in Pernicious Anemia: A Systematic Review. Cureus.

[126] Nozaki K., Ogawa M., Williams J.A., Lafleur B.J., Ng V., Drapkin R.I., Mills J.C., Konieczny S.F., Nomura S., Goldenring J.R. (2008). A Molecular Signature of Gastric Metaplasia Arising in Response to Acute Parietal Cell Loss. Gastroenterology.

[127] O’Neal R.L., Nam K.T., LaFleur B.J., Barlow B., Nozaki K., Lee H.-J., Kim W.H., Yang H.-K., Shi C., Maitra A. (2013). Human Epididymis Protein 4 Is Up-Regulated in Gastric and Pancreatic Adenocarcinomas. Hum. Pathol..

[128] Guo Y.-D., Wang J.-H., Lu H., Li X.-N., Song W.-W., Zhang X.-D., Zhang W.-M. (2015). The Human Epididymis Protein 4 Acts as a Prognostic Factor and Promotes Progression of Gastric Cancer. Tumor Biol..

[129] Piao J., Lee H.G., Kim S., Kim D., Han H., Ngo H., Park S., Woo J., Lee J., Na H. (2016). *Helicobacter pylori* Activates IL-6-STAT3 Signaling in Human Gastric Cancer Cells: Potential Roles for Reactive Oxygen Species. Helicobacter.

[130] Nakagawa H., Tamura T., Mitsuda Y., Goto Y., Kamiya Y., Kondo T., Wakai K., Hamajima N. (2013). Significant Association between Serum Interleukin-6 and *Helicobacter pylori* Antibody Levels among H. Pylori Positive Japanese Adults. Mediators Inflamm..

[131] Kim D.-K., Oh S.Y., Kwon H.-C., Lee S., Kwon K.A., Kim B.G., Kim S.-G., Kim S.-H., Jang J.S., Kim M.C. (2009). Clinical Significances of Preoperative Serum Interleukin-6 and C-Reactive Protein Level in Operable Gastric Cancer. BMC Cancer.

[132] Sánchez-Zauco N., Torres J., Gómez A., Camorlinga-Ponce M., Muñoz-Pérez L., Herrera-Goepfert R., Medrano-Guzmán R., Giono-Cerezo S., Maldonado-Bernal C. (2017). Circulating Blood Levels of IL-6, IFN-γ, and IL-10 as Potential Diagnostic Biomarkers in Gastric Cancer: A Controlled Study. BMC Cancer.

[133] Vainer N., Dehlendorff C., Johansen J.S. (2018). Systematic Literature Review of IL-6 as a Biomarker or Treatment Target in Patients with Gastric, Bile Duct, Pancreatic and Colorectal Cancer. Oncotarget.

[134] World Health Organization (2010). WHO Recommendations on the Diagnosis of HIV Infection in Infants and Children.

